# Ultrasonic Vitrectomy Performance Assessment Using Micro-Extensional Rheology

**DOI:** 10.1167/tvst.12.2.24

**Published:** 2023-02-15

**Authors:** John C. P. Hollister, Mercedes Rodriguez, Helia Hosseini, Asael Papour, Jean-Pierre Hubschman, H. Pirouz Kavehpour

**Affiliations:** 1 Department of Mechanical and Aerospace Engineering, University of California – Los Angeles, Los Angeles, CA, USA; 2 Stein Eye Institute, University of California – Los Angeles, Los Angeles, CA, USA; 3 Department of Bioengineering, University of California – Los Angeles, Los Angeles, CA, USA; 4 Bausch and Lomb, St Louis, MO, USA; 5 Stein Eye Institute, University of California – Los Angeles, Los Angeles, CA, USA; 6 Department of Mechanical and Aerospace Engineering, Department of Bioengineering, University of California – Los Angeles, Los Angeles, CA, USA

**Keywords:** extensional, rheology, ultrasonic, vitrectomy, vitreous

## Abstract

**Purpose:**

The purpose of this study was to assess the performance of ultrasonic (US) vitrectomy devices by quantifying and comparing its impact on extracted vitreous properties to conventional pneumatic blade (PB) cutters using micro-extensional rheology. US vitrectomy is a new technology that offers an alternative to PB cutters used in vitreo-retinal surgeries.

**Methods:**

Thirty-six porcine vitreous samples were extracted using US and PB cutters. Each sample was kept at 4°C and tested within 24 hours postmortem and 4 hours post-vitrectomy. A recently developed micro-extensional rheology technique is used to infer the relative protein fragment size of extracted vitreous by quantifying the extensional relaxation time.

**Results:**

US-extracted vitreous exhibited extensional relaxation times orders of magnitude lower than PB-extracted vitreous (0.37 ms and 27.25 ms, respectively). Relaxation time is directly correlated to the fragment size of the collagen fibers in the vitreous. The formation of beads-on-a-string droplets within the PB samples indicates the presence of larger collagen fragments. These droplets were not seen on US samples.

**Conclusions:**

This new micro-extensional rheology technique can identify significant differences in physical properties of extracted vitreous. Long relaxation times and beads-on-a-string droplets within the PB vitreous samples indicate larger protein fragments compared to the US samples.

**Translational Relevance:**

Higher fragmentation of vitreous and lower extensional relaxation times may improve retina safety due to a reduction in vitreo-retinal traction resulting from the continuous shear action and aspiration applied by ultrasonic vitrectomy technology.

## Introduction

Vitrectomy, or the surgical removal of vitreous, is a required step in most vitreo-retinal surgical procedures. Vitreous is a transparent gel-like substance found between the lens capsule and retina. It is composed of an inflated and highly hydrated collagen matrix with 99 wt.% water, 0.9 wt.% salts, and less than 0.1 wt.% collagen and hyaluronic acid.^1^^,^^2^ Vitreous is related to various functions, including maintaining transparency, protecting the retina from trauma, and other metabolic requirements.^3^ Due to the highly connected matrix of collagen fibers, vitreous removal can cause traction in areas of localized retinal adhesion.^4^ Thus, vitrectomy must temper extraction speed with the cutting action to maintain retina safety.^5^

**Figure 1.**
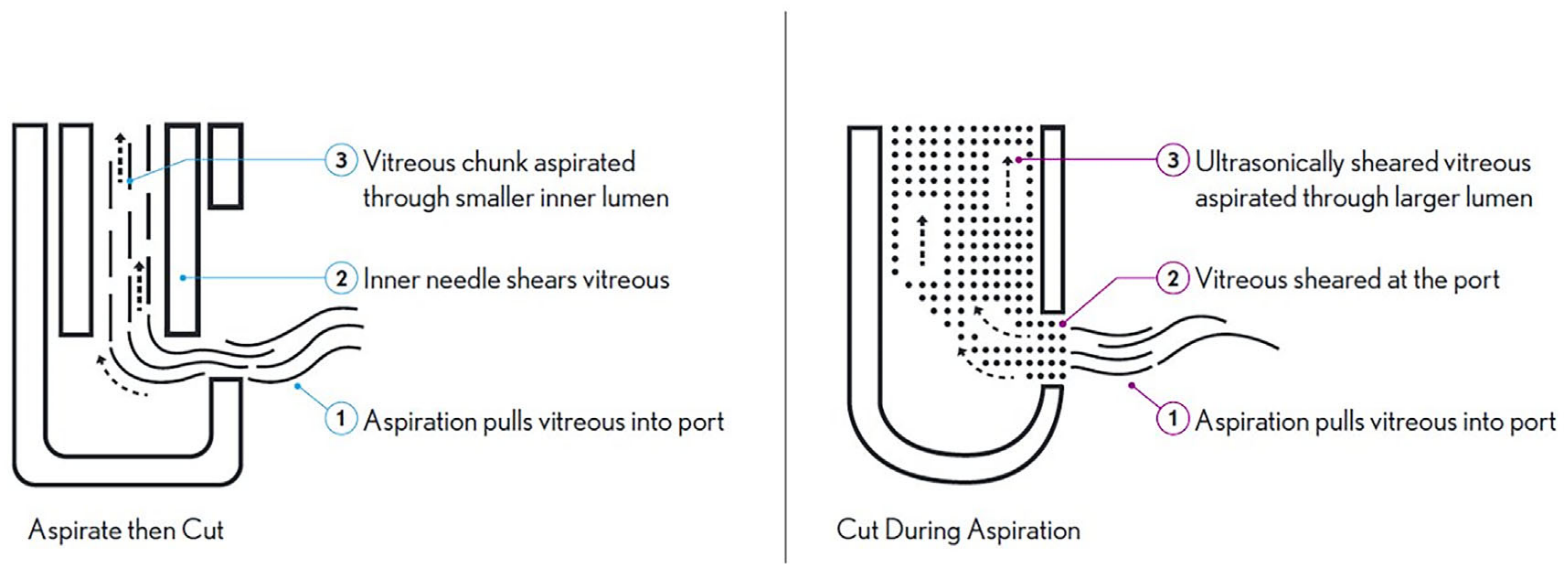
Comparison of pneumatic and ultrasonic mechanisms of action. (*Left*) Conventional pneumatic mechanisms aspirate intact vitreous and use inner needle blade to shear the tissue inside the needle lumen. (*Right*) Ultrasonic mechanisms shear the vitreous at port entrance, aspirating only ultrasonically sheared tissue. Figure courtesy of Bausch & Lomb.

Conventional pneumatic blade (PB) cutters use a “guillotine-style” cutter to aspirate vitreous out of the eye (Fig. 1). These cutters aspirate vitreous into the needle and then cut. This action creates traction where the retina may be pulled due to the aspiration forces.^4^ Ultrasonic (US) cutters, on the other hand, use a 100% open port design that continuously shears and aspirates the vitreous. The US energy is transmitted from the device's transducer to the needle probe. The port, located at the side of the needle near the distal end, vibrates at US frequencies enabling focused tissue cutting capabilities by the port walls.^6^^–^^8^ The two cutting technologies change the mechanical properties of the vitreous by breaking down the long collagen fibrils responsible for the mechanical properties of the gel-like vitreous.^9^ The PB cutter mechanism uses a series of discrete cutting steps where intact vitreous is aspirated and sheared at low relative speeds. Because the US mechanism allows for continuous, smooth aspiration of vitreous, less vitreous traction occurs with US cutters than PB cutters.^10^

Rheological study of the vitreous dates back to Lee and colleagues in 1992.^11^^–^^13^ Extensional rheology is a growing field where the properties of fluids are analyzed as they are subjected to extensional strain rather than the more typical shear strain.^14^ There are many applications where extensional strains are important, especially with biological materials like surgically extracted vitreous.^15^^–^^18^ The cross-linking and molecular interaction of polymers and proteins in solution cause fluids to exhibit non-Newtonian behaviors in extensional flow.^19^^,^^20^ These effects, however, cannot be identified with shear rheology.^21^ Therefore, performance assessment of vitrectomy technology using extensional rheology is ideal because extracted vitreous can be considered a non-Newtonian fluid containing dispersed collagen fibrils.

Extensional behavior of protein solutions is characterized by the formation of fluid structures where elastic protein interactions dominate the fluid flow.^22^ Lower molecular weight solutions exhibit relatively weak protein interaction, and purely Newtonian solutions, such as water and saline solution, show no interaction at all.^21^ It is hypothesized and observed that the US mechanism of action breaks collagen fibrils into much smaller fragments compared to the PB cutters.^23^ The purpose of this study is to quantify the extensional behavior of chopped vitreous and assess the mechanism of action of US vitreous cutters by comparing their performance to conventional PB cutters. To our knowledge, this is a first attempt at quantifying surgically extracted vitreous properties using extensional rheology technology.

## Methods

### Vitreous Extraction

Pig vitreous was used as a tissue analog for human vitreous, extracted from eyes using two different vitreous cutters: a PB cutter (BiBlade; Bausch & Lomb, St. Louis, MO) and a US cutter (Vitesse; Bausch & Lomb). Freshly harvested, unscalded, enucleated pig eyes were purchased from Sierra Medical Supplies (Whittier, CA). Preparation of each eye was performed under a surgical microscope (M840; Leica Microsystems, GmbH, Wetzlar, Germany). Eyes were acquired within 24 hours postmortem, and the tests were performed within 8 hours of receipt and 4 hours post-vitrectomy to ensure consistency in results. All eyes were stored at 4°C prior to the experiments.

The eyes underwent an open-sky pars plana vitrectomy while secured to a custom polystyrene holder. The procedures were conducted using the Bausch & Lomb (B&L) Stellaris PC Vision Enhancement System with hardware and software modifications to provide ultrasonic vitrectomy drive capability (Bausch & Lomb). A trocar was placed temporally 4 mm behind the limbus followed by a temporal corneal incision created with a 2.8 mm keratome blade. Surgical ophthalmic scissors were then used to remove the cornea by cutting through the corneal incision and following the edge of the corneoscleral limbus. Then the lens was squeezed out of the eye in one whole piece and the capsule was removed. Minimal iris manipulation was attempted to avoid contaminating the subsequent vitreous samples with pigment or other tissue fragments. Each probe was introduced via the pre-placed trocar to perform the vitrectomy and collect the undiluted chopped vitreous samples into sterile “BD Vacutainer Glass Serum” tubes (16 × 100 mm, 10.0 mL - no additives - Becton Dickson and Company, Franklin Lakes, NJ) using a vitreous trap technique.^24^ This technique was first described by Dr. Susan M. Malinowski in 2010, and it allows the surgeon to obtain an undiluted vitreous sample with aspiration in a controlled way, as well as diminishing the risk of contamination and providing a safe means to transfer.^24^ Special attention was paid to avoid contaminating the samples with pigment from the retina or the iris, and to avoid touching areas of detached mobile retina with the cutters. For this purpose, the vitrectomy was focused on the central vitreous rather than vitreous close to the retina or iris. Two samples were taken from each pig eye.

The dual-action 23- and 25-gauge PB probes were tested at 100, 300, and 600 mm Hg and at 15,000 cuts per minute (cpm). The 23- and 25-gauge US probes were tested at 60 µm stroke and at 100 and 300 mm Hg vacuum setting. Flowrate is the factor that determines the vitreous fragment size at a given cut rate. Due to the US cutter's open smaller port design and larger inner needle lumen, the flowrate at 300 mm Hg is comparable to the upper limit of the PB cutter achieved at 600 mm Hg (internal communication). Furthermore, the US cutter is not used in surgeries above 250 mm Hg. Therefore, conducting this experiment with US probes at usable vacuum ranges and comparable port flowrates was found to be adequate. The US cut rate is nominally 38 kHz and 41 kHz for the 23- and 25-gauge needles, respectively. Considering one cycle to be equivalent to one cut, these cutter frequencies correspond to cut rates of more than 2 million cpm for the US cutters.

### Micro-Extensional Rheology

**Figure 2.**
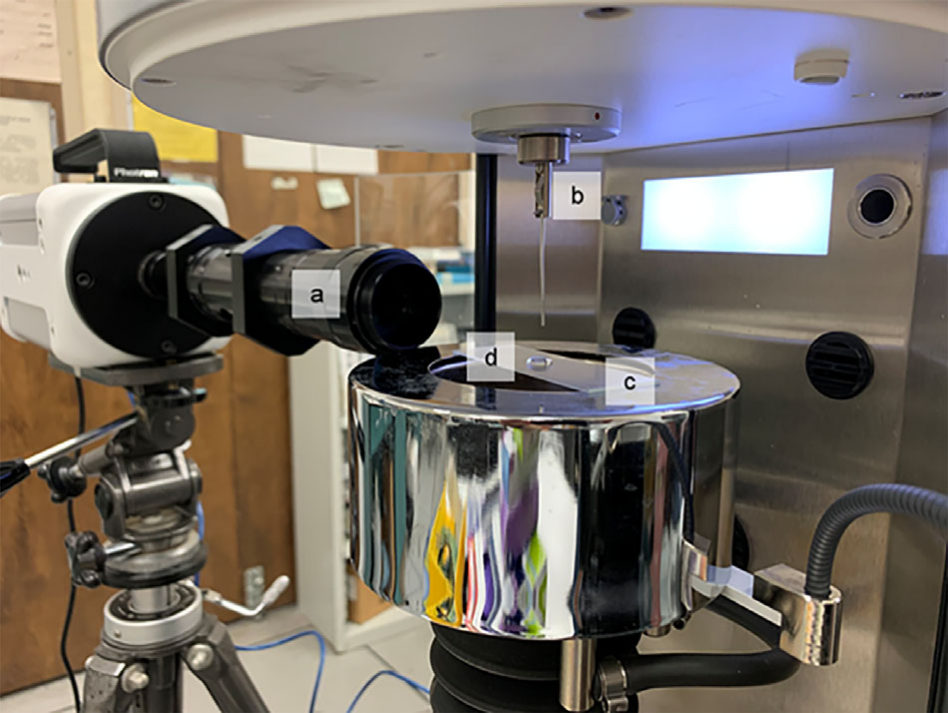
Micro-extensional rheology test apparatus. (**a**) Photron High Speed camera, (**b**) Kruss K-100 tensiometer with 1 mm platinum probe, (**c**) hydrophobically treated surface, and (**d**) liquid sample.

To analyze the extensional rheological properties of small samples, we adapted a simple rheometric method to apply a large strain rate to very small sample volumes.^25^ Sample volumes were typically between 50 and 100 microliters. Each was dispensed onto a hydrophobically treated substrate to create a domed droplet. Using a KRUSS tensiometer (K-100; KRUSS GmbH, Hamburg, Germany) in the Surface Tension setting, each sample droplet was penetrated with a 1 mm diameter platinum rod. The probe penetrated 0.5 mm into each sample. The probe was then quickly removed, so that the surface of the sample was perturbed upward. Each sample was tested independently three times. Testing was performed at room temperature, but each sample was kept at 4°C prior to testing (Fig. 2).

As the sample dripped from the probe to the rest of the droplet, an elasto-capillary liquid bridge formed between the probe and sample droplet. A high-speed camera (NOVA R2; PHOTRON USA INC., San Diego, CA) set to 8000 frames per second and 1/10,000 s^−1^ shutter speed captured the evolution of this liquid bridge. The high-speed images were converted to binary in ImageJ, and the evolution of the fluid structure over time was measured using an edge-finding program in Matlab.^26^ The exponentially decaying portion of these data were fit to the following relationship developed by Entov and Hinch.^27^

(1) $$\frac{R\left(t\right)}{R_{0}}={\left(\frac{G_{E}R_{0}}{2\sigma }\right)}^{1/3}\exp\left(-\frac{t}{3\lambda_{E}}\right)$$

From this model, the extensional relaxation time (λ_E_) of the elasto-capillary regime was calculated for each trial (see Fig. 4). Longer λ_E_ values correlate to greater cross-linking and higher molecular weight of proteins in the sample.^26^ Shorter relaxation times correlate to less cross-linking, which indicates shorter, lower molecular weight protein molecules are present in the sample.^26^

## Results

Thirty-six vitreous samples were successfully extracted from 20 pig eyes (2 samples per eye). Twelve samples were tested after extraction with the US cutter. Twenty-four samples were tested after extraction with the PB cutter (see the Table).

**Table.** Summary of Cutter Configuration and Resultant Extensional Relaxation Times in Milliseconds

Vitrectomy Method				λ_E_ (ms)	
	Number of Samples	Cutter Size (Gauge)	Vacuum Pressure (mm Hg)	Mean	Stdev
Ultrasonic	3	23	100	0.17	0.10
	3	23	300	0.39	0.10
	3	25	100	0.13	0.06
	3	25	300	2.34	2.46
(Total)	(12)			(0.37)	(1.50)
Pneumatic blade	4	23	100	25.79	12.95
	4	23	300	24.17	15.33
	4	23	600	37.63	21.17
	4	25	100	19.11	8.47
	4	25	300	23.47	12.41
	4	25	600	33.33	11.56
(Total)	(24)			(27.25)	(15.08)

Stdev, standard deviation.

**Figure 3.**
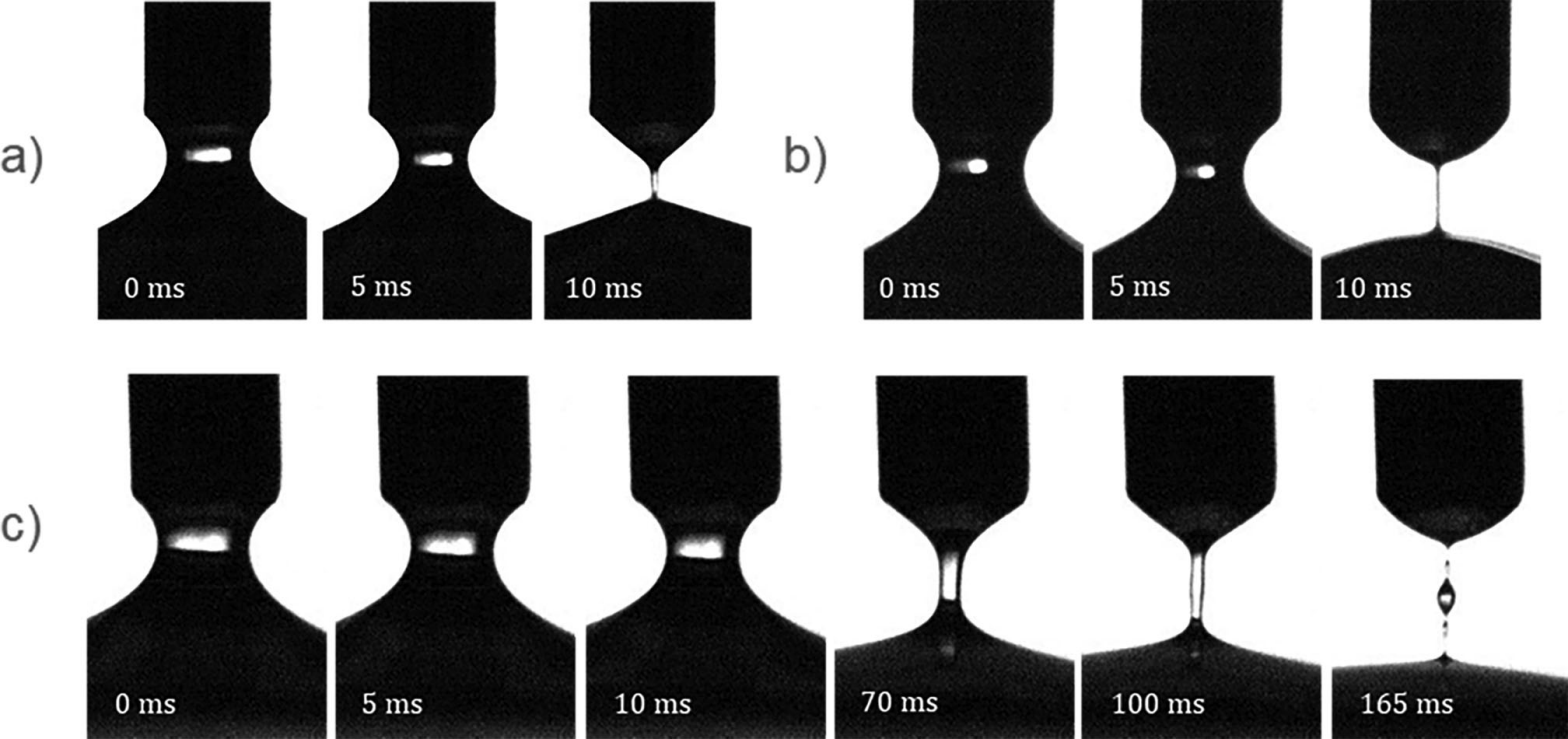
Radius evolution images starting at approximately 60% radius, taken 5 ms apart. (**a**) Balanced saline solution, containing no polymer or protein and showing no extensional relaxation behavior. (**b**) Vitesse ultrasonic cutter vitreous. (**c**) BiBlade pneumatic cutter vitreous at longer time steps. Beads-on-a-string phenomenon was observed at the end of the liquid bridge evolution (at 165 ms).

**Figure 4.**
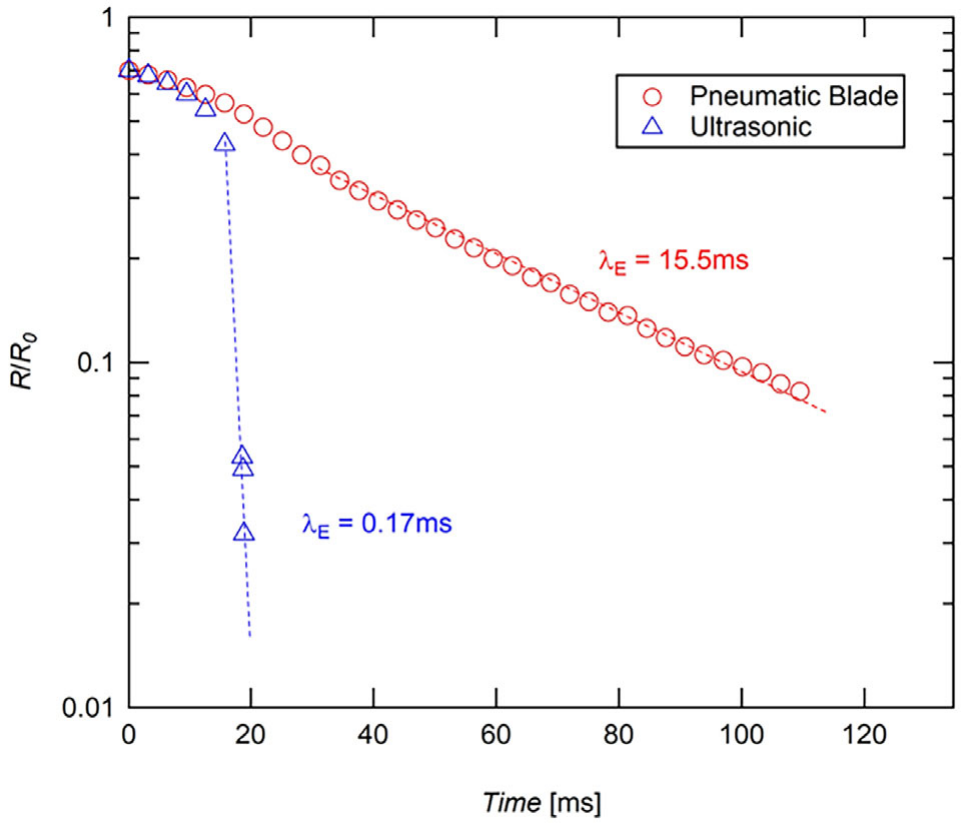
Radius evolution plots of representative US and PB samples. The elasto-capillary regime is identified with *dotted lines*. The first data points in the graphs represent the inertia-capillary regime governed by a power-law decay. Extensional relaxation time, λ_E_, is calculated by fitting Equation 1 to elasto-capillary regime.

The extensional rheology behavior varied dramatically between the US and PB cutter vitreous samples (Figs. 3, 4). The liquid bridge that forms between the two surfaces devolved in a few milliseconds with the US samples (see Fig. 3b). Qualitatively, this behavior is similar to balanced saline solution (BSS), a Newtonian fluid which contains no protein, and therefore does not exhibit elasto-capillary behavior^28^ (see Fig. 3a). The liquid bridge of the PB samples, on the other hand, persisted for more than 100 ms (see Fig. 3c).

Another characteristic difference between the PB and US samples was the beads-on-a-string structures that were only observed with the PB samples (see Fig. 3c; 165 ms). These structures were observed on each of the 24 PB samples but were not observed on any US sample. This phenomenon is an indication of large protein fragments being present in the sample.^29^ As the liquid bridge devolves, the fluid surrounding the protein structures evacuates the liquid bridge, leaving only a tangled mass of proteins. In several trials of this experiment, the beads-on-a-string structures persisted even after the fluid reached equilibrium. In these cases, the fluid bridge had to be manually removed before testing could resume.

### Analysis

**Figure 5.**
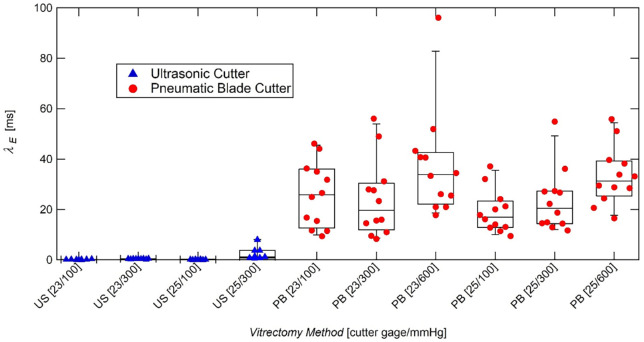
Box plots of sample relaxation times extracted using ultrasonic and pneumatic blade cutters. The average extensional relaxation time for US and PB samples were 0.37 ms and 27.25 ms, respectively.

Extensional relaxation times (Fig. 5) averaged 27.25 ms for the PB samples, and 0.37 ms for the US samples, a reduction of approximately 99%. The average extensional relaxation time for the US samples was significantly lower than the PB samples (*P* < 0.001) with up to two orders of magnitude in difference. The inertia-capillary regime of the US samples dominated the flow, and the elasto-capillary liquid bridge existed for only a few image frames in each trial, indicating the protein interactions had little effect on the flow in these samples. The relatively high extensional relaxation time for PB samples culminating in beads-on-a-string structures indicate the elasto-capillary effects of the protein interactions were significant. This is an indication of relatively large protein fragments and high cross-linking of the proteins within the samples.

Higher vacuum pressure resulted in significantly higher average extensional relaxation time in US 23- and 25-gauge samples (*P* = 0.004 and *P* = 0.018, respectively) and PB 25-gauge samples (*P* = 0.002). The vacuum pressure did not have a significant impact on the average relaxation time of the PB 23-gauge samples (*P* = 0.112).

Smaller needle gauges exhibited higher average extensional relaxation times for US samples at 300 mm Hg (*P* = 0.032), but not at 100 mm Hg (*P* = 0.548). For PB samples, the needle gauge did not significantly impact the average extensional relaxation time at any of the three vacuum pressures (100 mm Hg: *P* = 0.149, 300 mm Hg: *P* = 0.903, and 600 mm Hg: *P* = 0.543).

## Discussion

The macromolecular structure of the vitreous is dominated by a collagen fiber network inflated by hyaluronan and water. Pastor-Idoate et al. (2017) evaluated the US vitrector by qualitatively examining the histopathological changes in the vitreous after vitrectomy with US and PB vitrectors in pig cadaveric eyes, live swine, and human cadaveric eyes.^23^ Similar surgical outcomes were recorded with the two technologies. With US vitrectomy, however, finer fragmentation of the collagen and fewer long collagen fibrils from the residual collagen network was observed. Using this micro-extensional rheology technique, we have confirmed that the US cutting action results in much lower extensional relaxation times and therefore smaller protein fragments of the extracted vitreous can be inferred.

The effects of vacuum pressure and probe gauge on extensional relaxation time were outweighed by the effect of cutting method (US versus PB). Generally, it is expected that higher pressures, which correspond to higher flow rates,^7^ would result in coarser fragmentation of the vitreous and therefore higher extensional relaxation times. Our results are in partial agreement with this expectation, but in the case of the 23-gauge PB probe, the statistical significance was not strong. Similarly, it is expected that larger probe gauges would result in larger vitreous fragments and therefore higher extensional relaxation times. Our results showed the extensional relaxation times of the extracted vitreous were not strongly dependent on the probe gauge.

The results of this study support the findings of Pastor-Idoate et al. (2017), that the use of US cutters results in high fragmentation of the vitreous,^23^ which may allow for smaller needle gauges to be used during vitrectomy. PB cutters require a nontrivial amount of intact material to be aspirated by the needle, which requires needles to be relatively large.^7^ US cutters, however, fragment the vitreous prior to aspiration into small enough particles as to behave like water. Therefore, smaller needles may be used, which reduces the size of the entry wound during vitrectomy procedures with US cutters.

These results also indicate that many of the limitations identified by Stanga et al. (2017)^7^ can potentially be overcome by US cutting action. These limitations include undesirable turbulence at the cutter port and increased traction on the retina. The finer and continuous fragmentation of the US samples leading to lower extensional relaxation time may reduce the required vacuum pressure of these vitrectors. This has the potential to reduce risk of damage to the retina caused by the vacuum force during extraction.

Finally, the US action may show potential for the development of new tools and techniques. It can be utilized in a variety of geometries to improve ergonomics and promote safety. Because US cutters can effectively liquify the material around it, the results of this study also show promise that the technology can be applied to stiffer biological materials.

### Limitations

The goal of this paper was to quantify and assess the effect cutting method has on the rheological properties of extracted vitreous. To do so, we measured the extensional relaxation time, which is an indicator of the relative length of protein polymer fragments contained in a sample. The results presented here indicate that the US cutter chops vitreous into a much smaller fragments, as expected. However, this conclusion was not directly confirmed. If desired, the relative size of the proteins in samples of vitreous extracted using PB and US vitrectors can be directly confirmed using microscopy techniques like those used by Pastor-Idoate et al. (2017).^23^

## Conclusions

In summary, we have assessed ultrasonic vitrectomy technology and its performance compared to conventional PB vitrectors. By using extensional rheology, we were able to show significant differences in the vitreous samples extracted using the two cutter styles, where typical shear rheology was not likely to do so. When extracted using an ultrasonic cutter, the extensional relaxation times of the vitreous samples were orders of magnitude shorter compared to samples extracted using the pneumatic blade cutter (US sample mean = 0.37 ms, and PB sample mean = 27.25 ms). These results confirm that the US cutter breaks the vitreous collagen into very small fragments compared to pneumatic cutters. This has broad implications for improved ergonomics and safety of the ultrasonic cutter, as well as for the development and improvement of vitrectomy devices and techniques using US technology.
